# Supplementary material for: Bench‐to‐Bedside Translation of Self‐Healing Colloidal Hydrogels as Next Generation Design of Flowable Hemostatic Matrix: From Preclinical Evaluation to Human Clinical Trials
Source: Adv Sci (Weinh). 2026 Apr 29;13(40):e21713. doi: 10.1002/advs.202521713 (PMC13325802; doi:10.1002/advs.202521713)
Supplement: Supplementary file 1 — Supporting File: advs75039‐sup‐0001‐SuppMat.docx. [file ADVS-13-e21713-s001.docx]

Supporting Information

**Bench-to-bedside Translation of Self-Healing Colloidal hydrogels as Next generation Design of Flowable Hemostatic Matrix: From Preclinical Evaluation to Human Clinical Trials**

Ganjun Feng^a,^**^#^**, Kaiwen Chen^b,c,^**^#^**, Qiwei Ying^b,^**^#^**, Shuya Wang^b^, Xin Li^d^, Guanlin Li^d^, Yantao Zhao^e^, Binghua Ma^f^, Huanan Wang ^b,c,^*, Dawei Li^g,^*, Lijun Zhang^h,^*, Jian Zhang^f,^*, Yu Zhao^i,^*, Changle Ren^e,^*, Baodong Chen^j,^*, Yueming Song^a,^*

^a^ Department of Orthopedic Surgery and Orthopedic Research Institute & West China Hospital, Sichuan University, Chengdu, 610065, China

^b^ MOE Key Laboratory of Bio-Intelligent Manufacturing, Dalian Key Laboratory of Artificial Organ and Regenerative Medicine, School of Bioengineering, Dalian University of Technology, Dalian, 116024,China

^c^ State Key Laboratory of Fine Chemicals, Frontiers Science Center for Smart Materials Oriented Chemical Engineering, Dalian University of Technology, Dalian 116024, China

^d^ Shenzhen Huanova Biotechnology Co., Ltd, Shenzhen, 518104, China

^e^ Affiliated Central Hospital of Dalian University of Technology, Dalian, 116033, China

^f^ Department of Colorectal Surgery, Second Affiliated Hospital of Navy Medical University, Shanghai ,200003, China

^g^ Department of Otolaryngology, The Third People’s Hospital of Dalian, Affiliated to Dalian University of Technology, Dalian,116033, China

^h^ Department of Ophthalmology, The Third People’s Hospital of Dalian, Affiliated to Dalian University of Technology, Dalian,116033, China

^i^ Department of Orthopaedics, State Key Laboratory of Common Mechanism Research for Major Diseases, Peking Union Medical College Hospital, Chinese Academy of Medical Sciences and Peking Union Medical College, Beijing 100730, China

^j^ Department of Neurosurgery, Shenzhen hospital of Peking University, Shenzhen, 518036, China

*Corresponding author.

Email: [huananwang@dlut.edu.cn](mailto:huananwang@dlut.edu.cn) (Huanan Wang)

Email: [dw19770719@163.com](mailto:dw19770719@163.com) (Dawei Li)

Email: zhanglijun@dlut.edu.cn (Lijun Zhang)

Email: txzhangjian@126.com (Jian Zhang)

Email: zhaoyu6064@pumch.cn (Yu Zhao)

Email: changlerenle@dlut.edu.com (Changle Ren)

Email: chenbd@pkusz.edu.com (Baodong Chen)

Email: [hx_sym@163.com (Yueming](mailto:hx_sym@163.com(Yueming) Song)

**^#^** These authors contributed equally to this work

# Materials and Methods

## 1.1 Materials

CASH (Colloidose^®^) was supplied by Shenzhen Huanova Biotechnology Co., Ltd. (Batch No.: 20200302; specification: 0.5 g per unit). For preparation, 0.5 g of CASH was loaded into a 10 mL syringe. Sterile saline was drawn into a separate syringe and mixed with CASH through a Luer-lock connector using repeated back-and-forth extrusion until a homogeneous formulation was obtained. Final solid contents of 10%, 15%, and 18 % (w/v) were prepared and designated as CASH-10%, CASH-15%, and CASH-high, respectively. Air bubbles were removed prior to use to obtain uniform injectable colloidal gels. Surgiflo™ (flowable gelatin hemostatic matrix without thrombin; Johnson & Johnson Medical Devices Companies; Batch No.: 255422; specification: 8 mL per bottle) was used as the commercial control.

To determine the actual solid content of Surgiflo™, the product was prepared according to the manufacturer’s instructions by mixing with 2 mL of sterile physiological saline. The syringe was compressed repeatedly (10 cycles) to ensure complete mixing. An aliquot (1 mL) of the homogenized formulation was weighed to obtain the wet weight (m₁), freeze-dried, and reweighed to obtain the dry weight (m₂). The mass fraction was calculated as m₂/m₁, yielding an approximate solid content of 18%, which was designated as Surgiflo. For comparison with CASH at equivalent solid contents, diluted Surgiflo formulations with 10% and 15% (w/v) solid fractions were prepared by adding sterile saline and were denoted as Surgiflo-10% and Surgiflo-15%, respectively.

## 1.2 Characterizations

CASH and Surgiflo were directly subjected to scanning electron microscope (SEM, Nova Nano SEM 450) observation after freeze-drying to obtain their morphological characteristics. CASH and Surgiflo were respectively co-incubated with red blood cells and buffy coat for 1 hour, then fixed with 4% paraformaldehyde, dehydrated with graded ethanol solutions, and then the micro-morphology was observed by SEM after sputter-coating with gold.

Fourier transform infrared spectroscopy (FT-IR, Thermo Fisher Nicolet iS5) was used to analyze the chemical composition of CASH and Surgiflo. After freeze-drying, CASH and Surgiflo were ground into a fine powder and compressed into pellets for measurement. The spectral resolution was set at 4 cm⁻¹ with 32 scans collected per sample, and each sample was measured in triplicate to ensure reproducibility.

Nitrogen adsorption–desorption isotherms were obtained at 77 K (−195.85 °C) using a Micromeritics ASAP 2460 automated surface area and porosity analyzer. Approximately 0.15–0.19 g of each sample was loaded into analysis tubes and degassed under vacuum prior to measurement to eliminate surface-adsorbed contaminants.

The Brunauer–Emmett–Teller (BET) equation was applied to the adsorption branch of the isotherm to calculate the specific surface area within the appropriate relative pressure range. Total pore volume was calculated from the adsorption amount at P/P₀ ≈ 0.99. Micropore parameters were analyzed using the t-plot method, and average pore diameter was determined by the 4V/A (BET) model.

## 1.3 Mechanical Characterization

The rheological properties were performed using a Discovery Hybrid Rheometer (DHR, TA Instruments, USA) equipped with a parallel plate geometry (diameter: 20 mm). The temperature was maintained at 25 °C, and the gap distance was set to 1000 μm. The shear-thinning behavior was evaluated by measuring viscosity as a function of shear rate, which was varied from 1 s⁻¹ to 100 s⁻¹. After an initial time sweep at a strain of 0.5% and a frequency of 1 rad s⁻¹ for 4 min, an amplitude sweep was performed at a constant frequency of 1 rad s⁻¹ with strain increasing from 0.5% to 100%. Subsequently, the time sweep described above was repeated to assess the self-healing properties of the samples. All samples were subjected to three consecutive cycles of large-strain disruption and recovery. After each cycle, the recovery rate (%) of the storage modulus (G′), defined as the ratio of the recovered modulus during the time sweep to the initial modulus before high-strain deformation, was calculated to quantify self-healing efficiency. Frequency sweep measurements were conducted over a range of 0.1–100 rad s⁻¹ at a constant strain of 0.5% to characterize the viscoelastic properties.

For injection force measurements, all syringes containing CASH or Surgiflo samples were fixed vertically on a custom-designed scaffold in compression mode. A universal testing machine (E43, MTS Instruments, USA) was used to apply compression to the syringe plunger at a constant speed of 2 mm min⁻¹, and the corresponding force was recorded.

## 1.4 In vitro hemostatic activity determination

A custom-built burst pressure testing system was constructed using a pressure sensor coupled with a peristaltic pump. A 5 mm × 5 mm incision was made in fresh porcine skin, and the incision was sealed with CASH-10%, CASH-15%, and Surgiflo™, respectively. Blood was then injected at a constant flow rate of 0.1 mL s⁻¹ until leakage from the sealed incision was observed. The maximum pressure recorded immediately prior to leakage was defined as the burst pressure. Each sample was tested in quadruplicate. To assess the in vitro blood absorption capacity of the hemostatic gels, 0.5 mL of CASH and Surgiflo™ was placed onto pre-weighed filter paper. Fresh blood was continuously added until saturation was visually observed. The total mass after saturation was recorded, and the mass of blood absorbed by the filter paper alone was subtracted to calculate the net blood absorption capacity of each formulation. Each sample was tested six times (n = 6). Thromboelastography (TEG/ROTEM) was performed to investigate the in vitro coagulation performance. The samples were mixed with citrate-anticoagulated porcine whole blood (3.2% sodium citrate) at volume ratios of 1:5 and 1:10. After mixing, 300 μL of the sample–blood mixture was combined with 20 μL of 0.2 M CaCl₂ to reintroduce calcium ions and initiate coagulation. An intrinsic pathway activator (Factor XII) was added according to the manufacturer’s protocol, and clotting parameters were recorded automatically. Blood was collected from the auricular vein of Japanese rabbits and stored in sodium citrate anticoagulant tubes. The blood was centrifuged at 275 × g for 10 min, and platelet-rich plasma (PRP) was obtained. PRP was mixed with platelet-poor plasma (PPP) to standardize platelet concentration. Approximately 0.2 g of each sample was placed in a centrifuge tube and immersed in 0.5 mL of platelet-containing plasma, followed by incubation at 37 °C for 10 min. The samples were subsequently dehydrated using graded ethanol solutions (20%, 40%, 60%, 80%, and 100%), fixed with 4% paraformaldehyde, freeze-dried, sputter-coated with gold, and observed by scanning electron microscopy (SEM) to evaluate platelet adhesion and morphology.

## 1.5 Cytotoxicity evaluation of CASH

MC3T3-E1 preosteoblast cells were used to evaluate the cytocompatibility of the CASH hydrogel. Surgiflo™, due to its rapid disintegration in aqueous media, was unsuitable for direct surface cell culture and was therefore not included in this assay.

Cells were cultured in Dulbecco’s Modified Eagle Medium (DMEM, high glucose) supplemented with 10% fetal bovine serum (FBS) and 1% penicillin–streptomycin. CASH hydrogels were prepared as cylindrical discs (5 mm in diameter and 2 mm in thickness) and placed in 12-well plates. MC3T3-E1 cells were seeded onto the hydrogel discs at an appropriate density in 500 μL of complete medium per well. The cultures were maintained at 37 °C in a humidified incubator containing 5% CO₂ for 1, 3, and 5 days. Cell viability was quantified using the CCK-8 assay according to the manufacturer’s instructions. All experiments were performed in six replicates (n = 6), and the mean values were calculated. Cells cultured in complete medium without hydrogels served as the control group. Cell viability on CASH discs was further evaluated using the LIVE/DEAD® Viability/Cytotoxicity Kit (Invitrogen, Shanghai, China). Briefly, samples were incubated with 2 μM calcein-AM and ethidium homodimer-1 for 10 min at 37 °C. The hydrogel discs were then washed three times with culture medium, and live/dead cells were visualized using a laser scanning confocal microscope. To observe cell morphology and cytoskeletal organization, F-actin was stained with Alexa Fluor 594 Phalloidin (Invitrogen, USA), and nuclei were counterstained with DAPI (Solarbio, China). Samples collected on days 1 and 7 were fixed overnight in 4% paraformaldehyde (PFA), permeabilized with 0.1% Triton X-100 for 20 min, and blocked with 1% bovine serum albumin (BSA) for 45 min. After washing with PBS, samples were incubated with Alexa Fluor 594 Phalloidin for 45 min, followed by DAPI staining for 10 min. Fluorescence images were captured using a laser scanning confocal microscope.

## 1.5 In vitro and in vivo degradation of CASH

The degradation behaviors of CASH gels were accessed through an enzymatic degradation process. CASH gels (1 mL) were incubated in freshly prepared collagenase I solution (500 ng/mL) in PBS at 37 °C. At predetermined time points, the samples were exhaustively washed using deionized water, then freeze-dried and weighed to calculate the remaining mass percentage (n = 3). enzymatic degradation

For in vivo degradation，Colloidose^®^ (10% w/v, clinically relevant formulation) and Surgiflo™ were prepared under sterile conditions according to the procedures described above. For implantation, materials were loaded into sterile syringes and directly injected into the subcutaneous space. Male Sprague–Dawley rats (8 weeks old, 220–250 g) were anesthetized with isoflurane. After shaving and disinfecting the dorsal area, a small longitudinal skin incision (approximately 1 cm) was made. Blunt dissection was performed to create subcutaneous pockets on either side of the incision. Subsequently, 0.3 mL of Colloidose^®^ or Surgiflo™ was injected into the subcutaneous space using a sterile syringe. The injection site was gently massaged to ensure localized deposition of the material. The skin incision was closed using absorbable sutures. Animals were randomly assigned to different observation time points (1 week, 4 weeks, and 12 weeks). Throughout the study period, animals were monitored daily for general health condition, wound healing, and body weight changes. No signs of infection, abnormal behavior, or significant weight loss were observed. At predetermined time points, animals were euthanized, and the implantation sites together with surrounding tissues were carefully excised. The harvested specimens were fixed in 4% paraformaldehyde, embedded in paraffin, and sectioned for histological analysis. Hematoxylin and eosin (H&E) staining was performed to evaluate material resorption and local tissue response, and Masson’s trichrome staining was used to assess collagen deposition and tissue remodeling. Quantitative morphometric analysis was conducted to determine the residual material area fraction and the extent of tissue integration.

**1.6 Sustained Drug Release and Skin Wound Healing**

The drug release kinetics of Ciprofloxacin loaded on Colloidaose-10%, CASH-15% and Surgiflo were determined at a pH of 7.4. Ciprofloxacin (CIP) was dissolved in sterile saline and incorporated into the matrix (10 mg per 0.5 g gel). Release was performed in phosphate-buffered saline (PBS, pH 7.4) at 37 °C. Drug concentration was quantified using UV-Vis spectrophotometry at λmax = 272 nm, based on a pre-established calibration curve (R² > 0.99). Absorbance was recorded in triplicate at each time point for up to 20 days. Doxorubicin hydrochloride (DOX) was dissolved in sterile saline and loaded at 5 mg per 0.5 g matrix. Release was conducted in PBS (pH 7.4) at 37 °C under shaking. DOX concentration was determined by fluorescence spectrophotometry (Ex 480 nm, Em 590 nm) or UV–Vis at 480 nm. Calibration curves were prepared in PBS. Because DOX is light-sensitive, all experiments were performed under light-protected conditions. Dexamethasone (DEX), due to its hydrophobic nature, was dissolved in a small volume of ethanol (≤1%) before mixing with the hydrogel. Release was performed in PBS containing 0.1% Tween 80 to maintain sink conditions. Drug concentration was quantified by HPLC using a C18 column with UV detection at 242 nm. The mobile phase consisted of acetonitrile/water (60:40 v/v) at 1 mL/min. EGF (10 μg/mL) was incorporated into the gel immediately prior to testing. Release was carried out in PBS containing 0.1% bovine serum albumin (BSA) to prevent protein adsorption to the vessel wall. EGF concentration was quantified using a commercial ELISA kit according to manufacturer instructions. Because EGF is temperature-sensitive, release medium was pre-warmed and sampling was performed gently to avoid protein denaturation.

A total of 12 healthy male Sprague–Dawley (SD) rats (8 weeks) were randomly assigned to three groups (n = 4 per group): SHAM (untreated control), CASH, and CASH @EGF. Animals were maintained under specific pathogen-free conditions with a 12 h light/dark cycle at 25 °C and had free access to food and water. Recombinant epidermal growth factor (EGF) was dissolved in sterile saline to obtain a 10 μg/mL stock solution and incorporated into Colloidose immediately prior to application. Two symmetrical full-thickness excisional wounds (8 mm in diameter) were created on the dorsal region using a sterile biopsy punch. The excision extended through the panniculus carnosus to ensure a standardized full-thickness skin defect. Immediately after wound creation, 0.3 mL of the assigned formulation was applied topically to each wound. The SHAM group received no material treatment. Digital images were captured daily from Day 0 to Day 14 using a fixed imaging setup with a metric ruler included for calibration. Wound areas were quantified using ImageJ software by manually outlining the wound margins. The percentage of wound closure was calculated as:

$$Wound closure \left( \% \right)=\left( 1-\frac{A_{t}}{A_{0}} \right)\times100\%$$

where *A*_0_ represents the wound area on Day 0 and *A*_t_ represents the wound area at each designated time point. On Day 14, animals were euthanized and wound tissues were harvested for histological evaluation. Sections were stained with hematoxylin and eosin (H&E) to assess overall tissue architecture and re-epithelialization, and with Masson’s trichrome to evaluate collagen deposition and extracellular matrix remodeling.

## 1.7 In Vivo Hemostatic Evaluation

**In Vivo Hemostatic Assessment in Mouse Liver Model**

The in vivo hemostatic performance of CASH and Surgiflo was evaluated using a mouse liver puncture bleeding model. All animal experiments were approved by the Institutional Review Board of Dalian University of Technology. Male Sprague-Dawley (SD) rats (8 weeks old, 25-30 g) were used for the bleeding model. Rats were anesthetized with isoflurane and placed on a surgical board. The liver was exposed through an abdominal incision, and surrounding tissue fluid was removed using gauze. A pre-weighed filter paper was placed directly beneath the liver, separated from the abdominal incision by plastic wrap. A 10 mm long and 1.5 mm deep wound was created on the liver using a scalpel. Either CASH-10%, CASH-15%, or Surgiflo™ was immediately applied to the wound. The SHAM group received no hemostatic treatment and was left to coagulate naturally. After bleeding stopped, the absorbed blood was weighed, and the bleeding time was recorded. Each group contained 5 rats. Tissue samples were collected on Day 7 and Day 28 for histological analysis. Hematoxylin and eosin (H&E) staining was performed to examine the morphology of the wound tissue, and immunofluorescence staining for CD3 and CD68 was performed to evaluate the inflammatory response.

**In Vivo Hemostatic Assessment in Bama Minipig Model**

Bama minipigs (5 males and 16 females, weighing 20-30 kg, 6-12 months old) were provided by Wujiang Tianyu Biological Technology Co., Ltd. The animals were kept under a 12-hour light/dark cycle with unrestricted access to food and water at a temperature of 25°C. The experiments were approved by the Biological and Medical Ethics Committee of Dalian University of Technology. Minipigs were divided into three groups: SHAM (n = 3), Surgiflo™ (n = 6), and CASH (n = 12). The minipigs were pre-treated with scopolamine (0.01 mg/kg) to reduce respiratory secretions. After anesthesia induction using isoflurane, intravenous access was established. The liver was exposed by disinfecting the surgical site, and a 3.0 cm long, 0.5 cm deep incision was made on the liver. The SHAM group received no hemostatic treatment, and bleeding was controlled by manual compression for 5 minutes. In the Surgiflo™ and CASH groups, after 2 seconds of bleeding, the surface blood was gently wiped off with sterile gauze, and the hemostatic gel was applied to the incision. Hemostasis was observed every 20 seconds until complete cessation of bleeding. Product-tissue adhesion was subjectively evaluated. After surgery, cefazolin sodium (2.0 g) was intramuscularly injected twice daily for 3 days to prevent infection. Behavioral observations, including signs of discomfort, activity level, body temperature, local irritation, glandular secretion, fecal characteristics, and food intake, were recorded daily post-operation.

Blood biochemistry and coagulation profiles were measured pre- and post-operation, with statistical analysis performed to compare results. Upon sacrifice of the minipigs, anatomical examinations were conducted to identify any complications such as allergies, infections, hematoma formation, coagulation disorders, or adhesions. At 4 weeks post-operation, one minipig from each group was randomly selected for pathological analysis. Tissue samples from the liver, kidney, heart, lungs, spleen, and brain were collected, and frozen sectioning was performed on the liver incision block. The samples were stained with H&E and examined using a stereoscope microscope.

Table.S1 Number of animals at the different observation point

| Groups | Time | | |
| --- | --- | --- | --- |
|  | 1 week | 2 weeks | 4 weeks |
| Sham-operated | N/A | N/A | 3 |
| Surgiflo | 3 | N/A | 3 |
| CASH | 3 | 3 | 6 |

(Plus: N/A: Not Applicable, 'not applicable 'means' this column (for this group) is not applicable')

**Neurosurgical Cortical Hemorrhage Model and Hemostatic Evaluation Protocol**

For the neurosurgical hemostasis model, the scalp and fascia were incised along the cranial midline, and the cranial muscles were bluntly dissected. After removal of the periosteum, a bone window (~3 × 3 cm) was created in the parietal skull. The dura mater was opened while avoiding the superior sagittal sinus, and the cerebral cortex was exposed. A 1.0-1.5 cm cortical incision perpendicular to the superior sagittal sinus was made, and local vessels were transected to establish the bleeding model. After 5 s of free bleeding, blood loss over the next 10 s was collected using pre-weighed sterile gauze. The test material (1.5 mL) was then applied to the cortical defect, with additional material administered as needed up to a maximum of 3 mL. Hemostasis was monitored continuously, and time to hemostasis was recorded. Hemostasis was defined as the absence of rebleeding within 5 min. If bleeding persisted after 5 min, rescue hemostasis was performed using sterile gauze compression or electrocautery. In the blank control group, no hemostatic material was applied, and bleeding was allowed for 5 min before gauze compression. After hemostasis, the implanted volume was recorded, and the dura mater, bone flap, and overlying tissues were closed in layers.

## 1.8 Statistical analysis

In this study, rheological tests, in vitro hemostasis and coagulation assays, 2D cell culture experiments, and drug release experiments were typically performed in triplicate unless otherwise stated. The sample size (n) for each experiment is indicated in the corresponding figure legends or method descriptions. Animal experiments, human experiments, and other relevant assays were conducted according to the experimental design described above. Prior to statistical analysis, datasets were inspected for obvious outliers and evaluated for approximate normal distribution. No additional data transformation or normalization was applied unless otherwise specified. All data are presented as mean ± standard deviation (SD). For comparisons between two groups, an independent two-tailed Student’s t-test was used. For comparisons among three or more groups, one-way analysis of variance (one-way ANOVA) followed by Tukey’s post-hoc multiple comparison test was applied. The assumptions of normality and homogeneity of variance required for parametric tests were evaluated prior to analysis. All statistical analyses were performed using GraphPad Prism (version 9.0.0). A two-tailed P value < 0.05 was considered statistically significant. Statistical significance was denoted as *P < 0.05, **P < 0.01, and ***P < 0.001. Exact P values are reported in the corresponding figures or tables when applicable.


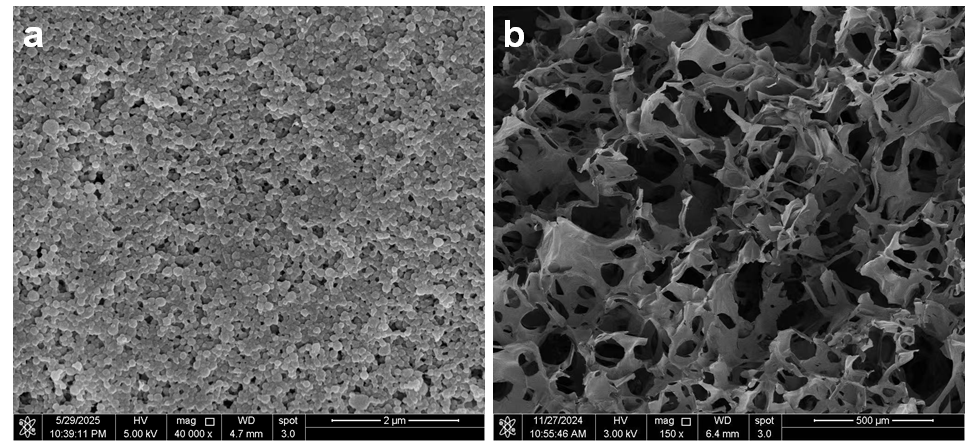


Figure S1. Representative scanning electron microscopy (SEM) images of (a) Colloidose^®^ and (b) Surgiflo™ after gel preparation and freeze-drying. Colloidose^®^ exhibits a densely packed nanoparticle-based microstructure with uniform submicron spherical particles (scale bar: 2 μm), whereas Surgiflo™ displays a porous, irregular microparticle network with large interconnected voids (scale bar: 500 μm), highlighting the distinct structural architectures of the two flowable hemostatic matrices.


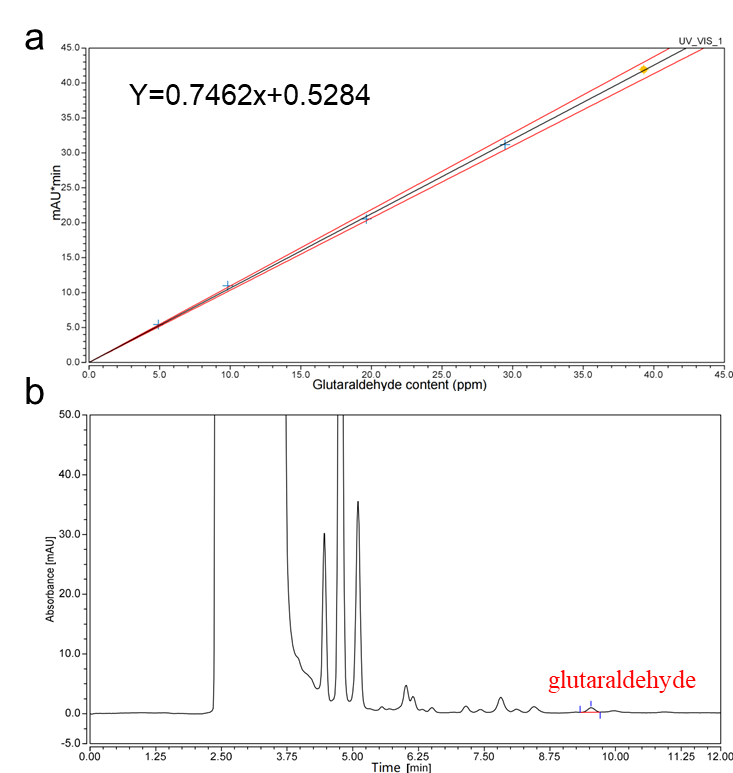


Figure S2. (a) Calibration curve of glutaraldehyde standard solutions obtained by high-performance liquid chromatography (HPLC), showing a linear relationship between peak area and glutaraldehyde concentration within the tested range. (b) Representative HPLC chromatogram of the final CASH gel formulation for determination of residual glutaraldehyde. Based on the calibration curve, the average residual glutaraldehyde content in the finished product was calculated to be 1.4 ppm, which is well below commonly reported safety thresholds for glutaraldehyde-crosslinked biomaterials.


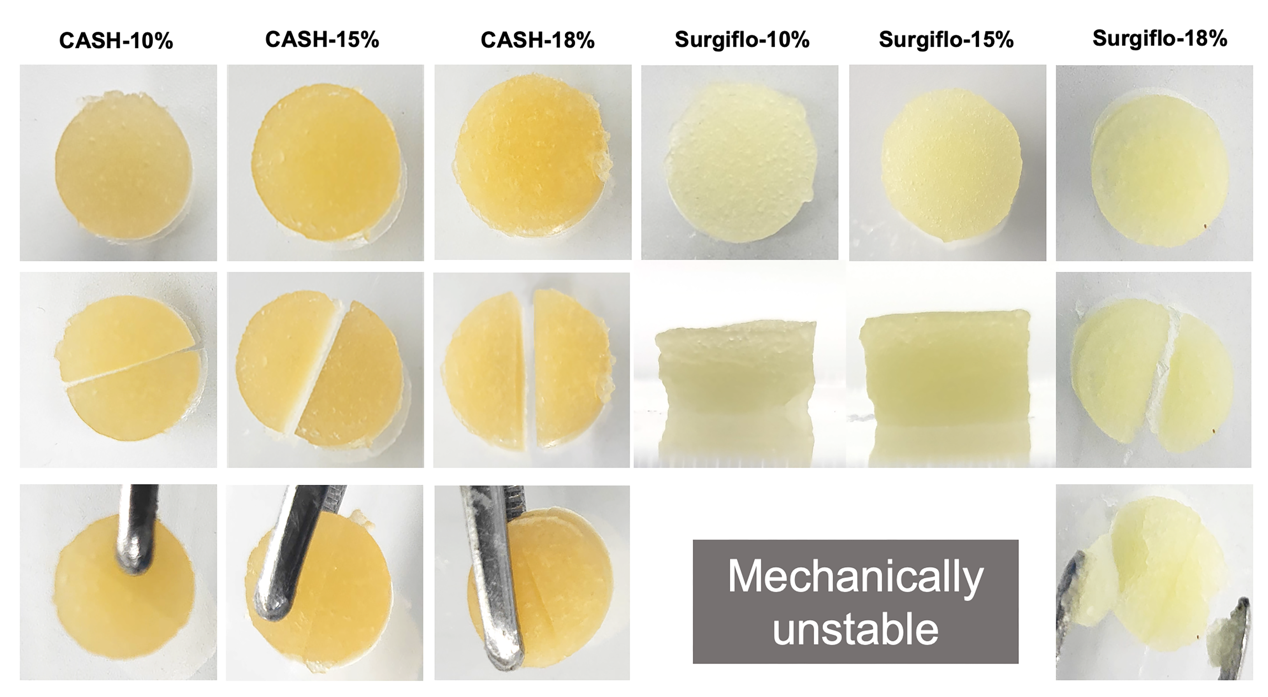


Figure S3. Macroscopic self-healing behavior of Colloidose^®^ and Surgiflo™ flowable matrices. Representative images showing structural recovery after mechanical sectioning. Colloidose^®^ discs were cut into halves and subsequently brought into contact, where they rapidly re-established a continuous, integrated structure, demonstrating efficient macroscopic self-healing. In contrast, Surgiflo™ exhibited limited structural reconstitution after sectioning, indicating weaker dynamic network recovery under identical conditions.





Figure S4. In vitro degradation of CASH gel (10 w/v% solid content) upon incubation in collagenase-containing PBS (n=3). The degradation rate increased progressively over time, with accelerated mass loss observed after Day 7 and near-complete degradation by Day 28, indicating enzymatically responsive biodegradability of the colloidal network.


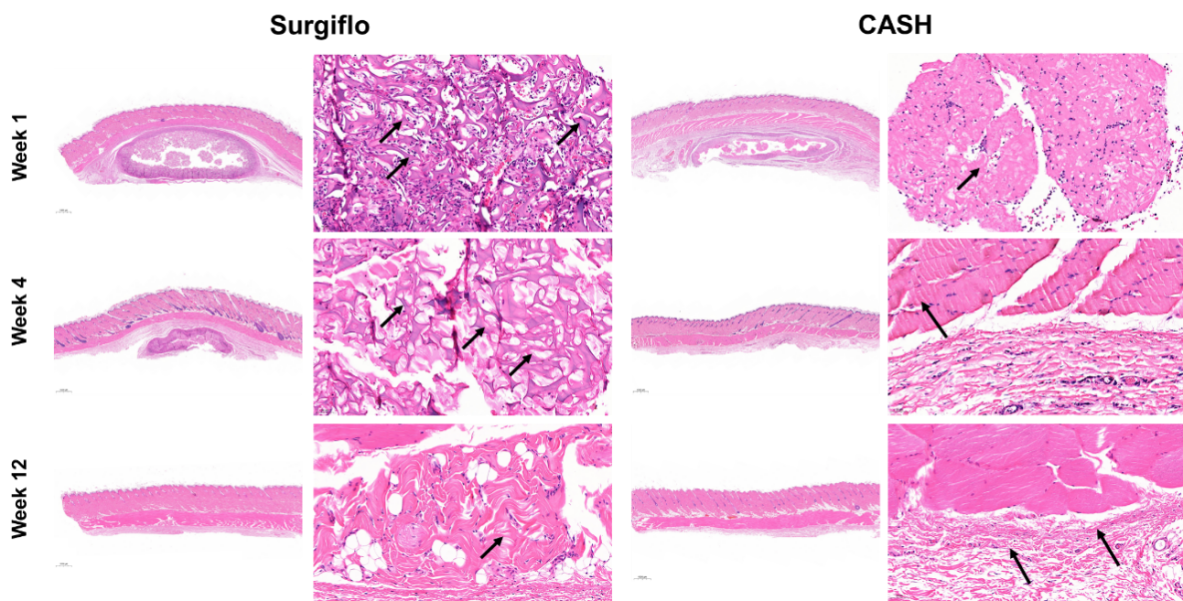


Figure S5. Representative H&E-stained sections of subcutaneous implantation sites at Weeks 1, 4, and 12. At Week 1, both materials were clearly identifiable with mild inflammatory cell infiltration (arrows). By Week 4, the CASH group showed markedly reduced residual material and increased host tissue integration, whereas visible fragments persisted in the comparator group. At Week 12, no obvious residual material was observed in either group, and tissue architecture appeared largely restored, indicating progressive in vivo degradation and favorable remodeling over time.


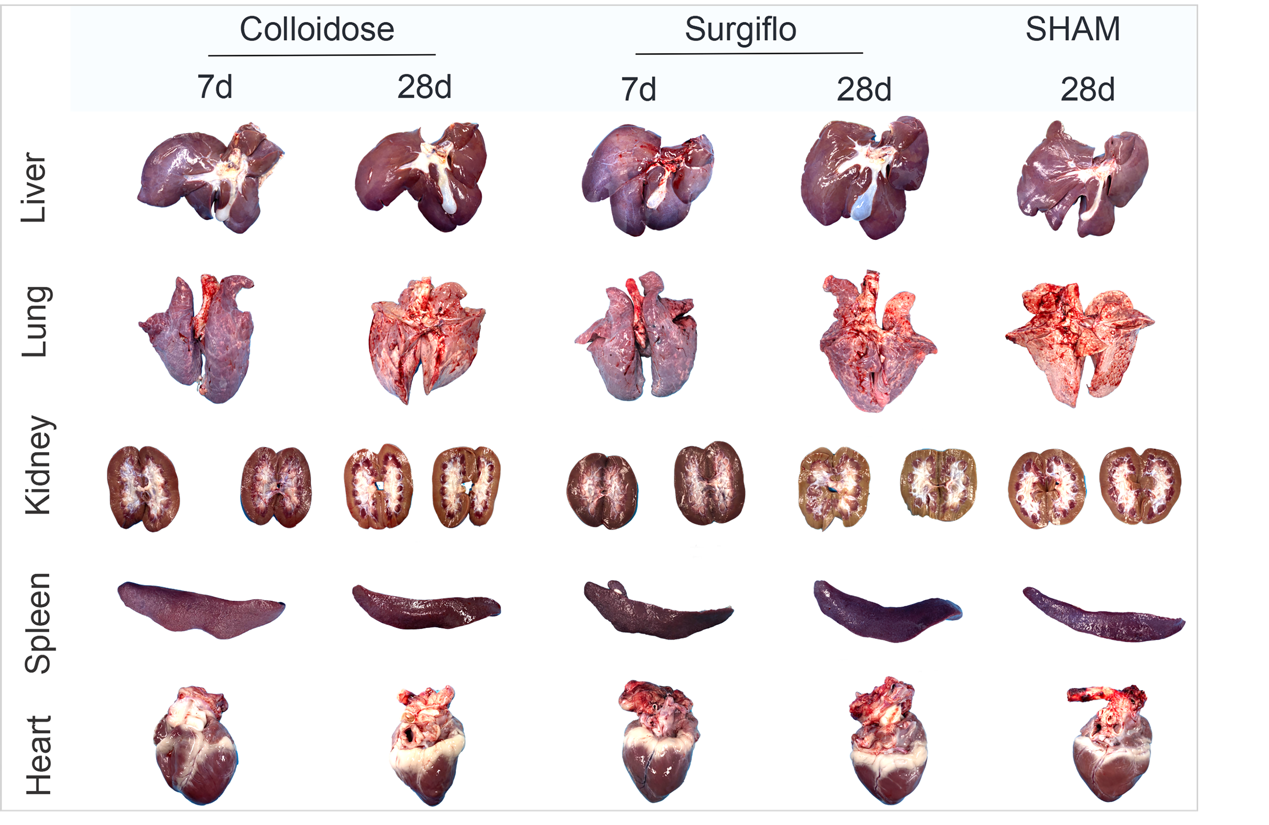


Figure S6. Representative gross images of major organs harvested from Bama minipigs at 7 and 28 days postoperatively following liver injury and treatment with Colloidose^®^ or Surgiflo™ flowable matrices. Organs shown include liver, lung, kidney, spleen, and heart. The SHAM group received injury without biomaterial application. No visible signs of abnormal enlargement, necrosis, hemorrhage, or pathological lesions were observed in any major organ across groups at the indicated time points, suggesting good systemic biocompatibility of both hemostatic matrices.


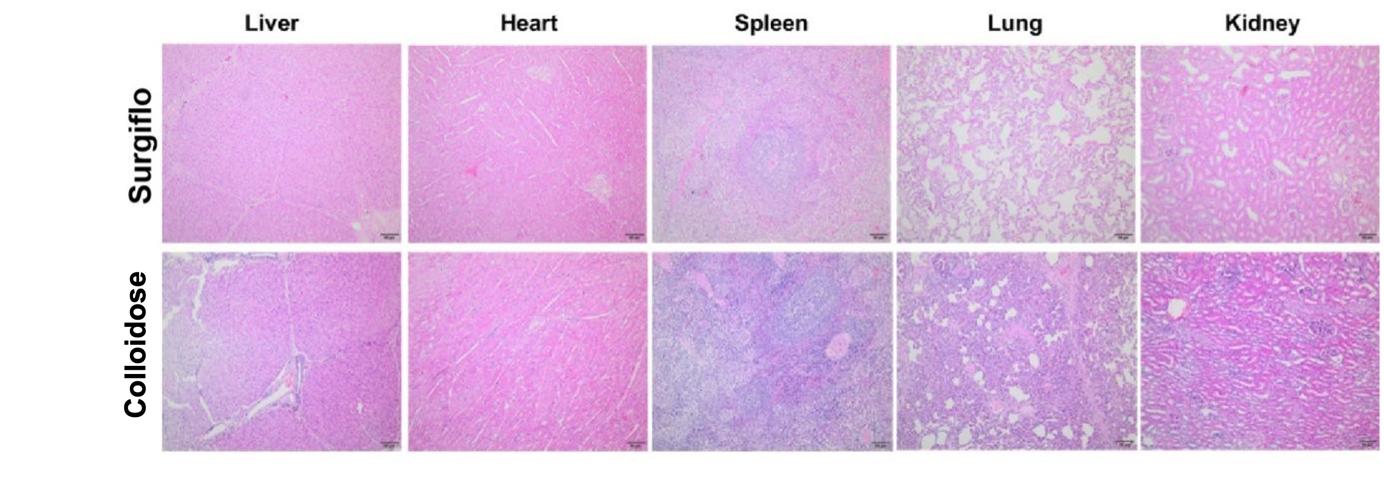


Figure S7. Histopathological evaluation of major organs (liver, heart, spleen, lung, and kidney) harvested from Bama minipigs at 1 week post-operation. H&E-stained sections were obtained from tissues distant from the surgical site to assess potential systemic toxicity or off-target inflammatory responses following treatment with Colloidose^®^ or Surgiflo™. No evident pathological abnormalities, inflammatory infiltration, or structural damage were observed in either treatment group.


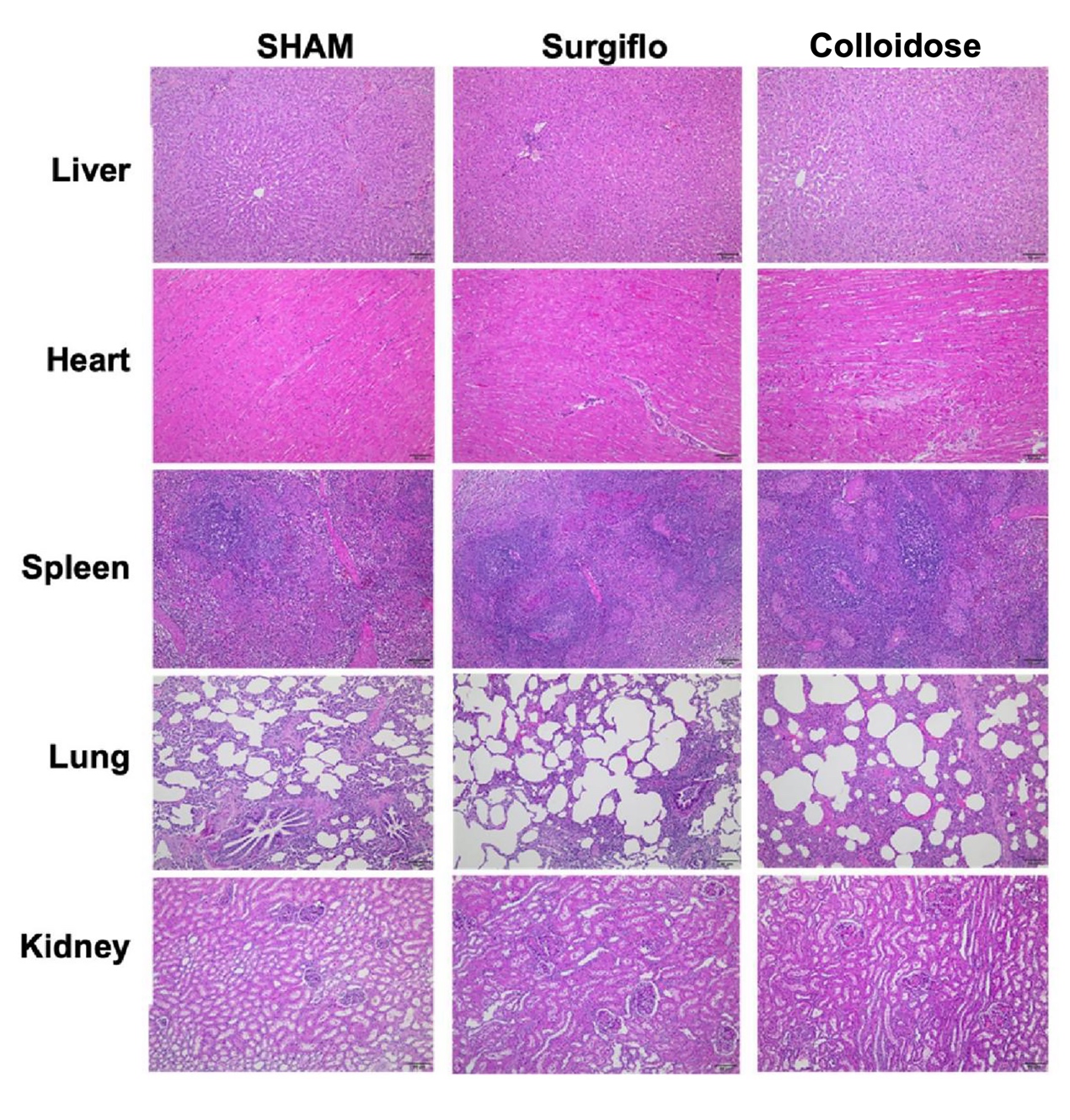


Figure S8. Representative H&E-stained sections of major organs (liver, heart, spleen, lung, and kidney) harvested from Bama minipigs at 4 weeks post-operation. Tissues were collected from regions distant from the surgical site to assess potential systemic toxicity or off-target pathological changes. No evident inflammatory infiltration, structural abnormalities, necrosis, or fibrosis were observed in the Colloidose^®^ or Surgiflo™ groups compared with the SHAM control, indicating the absence of detectable systemic adverse effects.


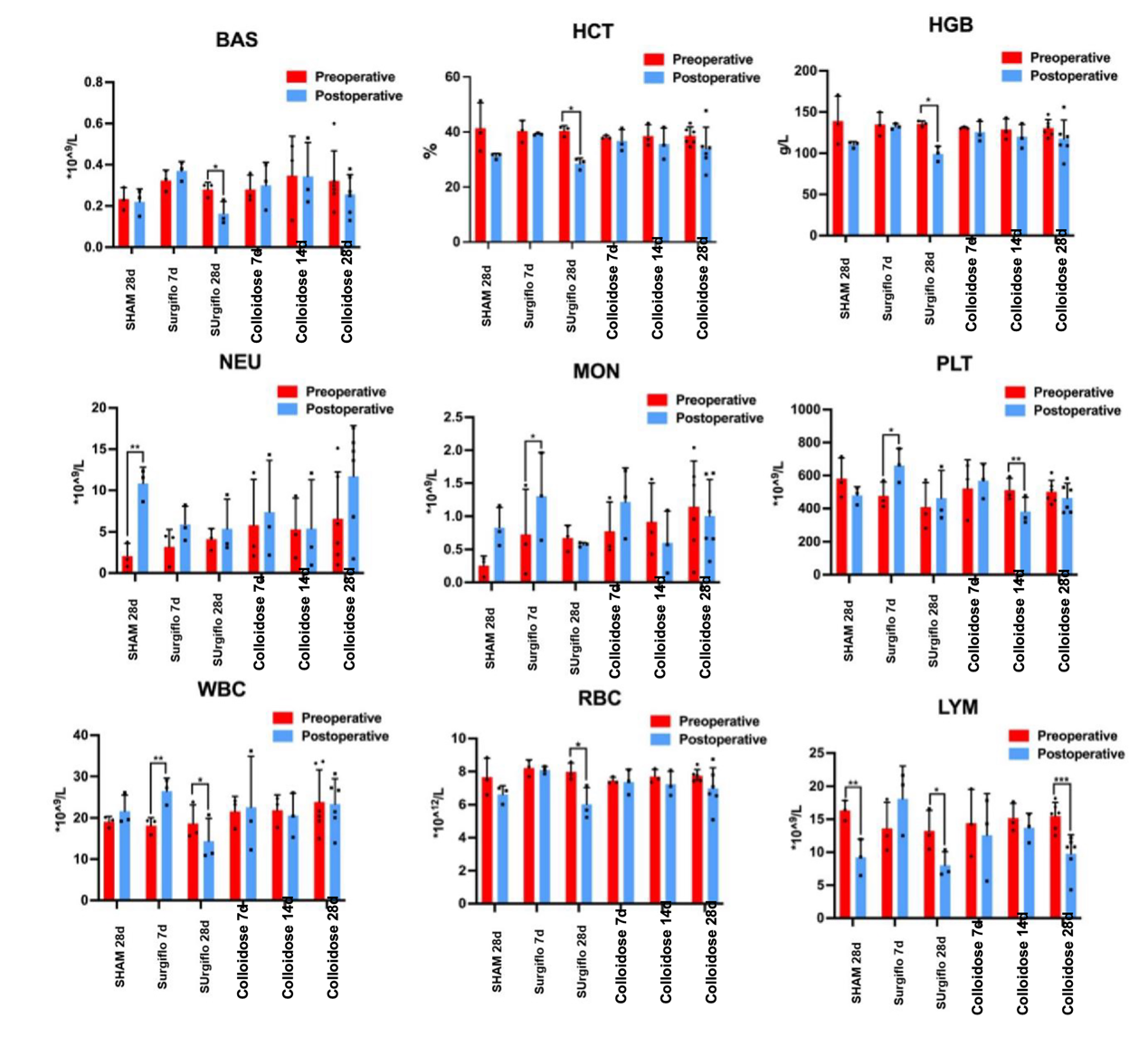


Figure S9. Routine hematological parameters in Bama minipigs measured before surgery (Preoperative, blue) and at necropsy (Postoperative, red) across different treatment groups. Evaluated indices included BAS (basophils), NEU (neutrophils), MON (monocytes), LYM (lymphocytes), WBC (white blood cells), RBC (red blood cells), HGB (hemoglobin), HCT (hematocrit), and PLT (platelets). No statistically significant differences were observed between preoperative and postoperative values in any group, indicating stable systemic hematological profiles and supporting the favorable blood compatibility of both Colloidose^®^ and Surgiflo™.


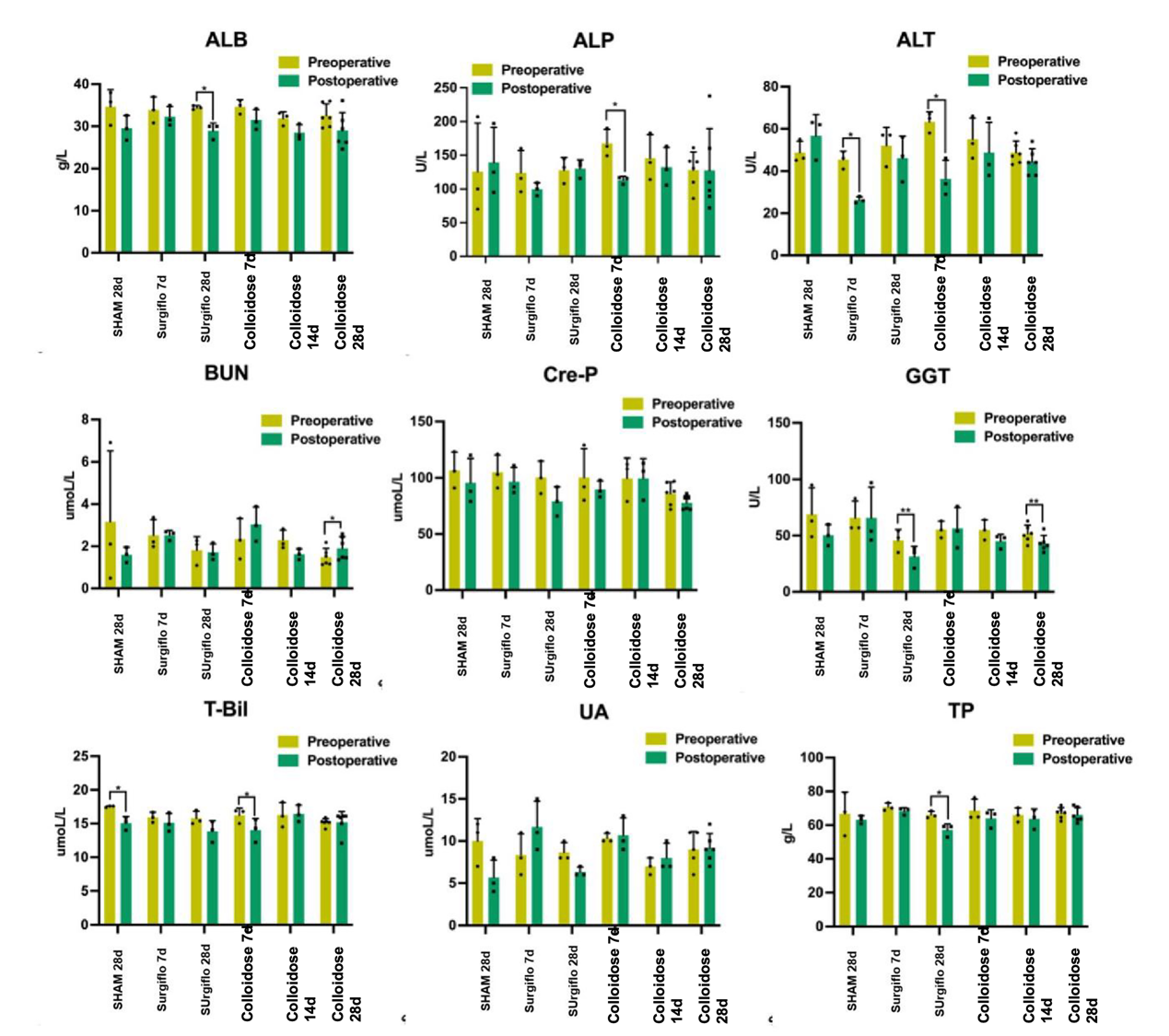


Figure S10. Blood biochemical parameters in Bama minipigs measured before surgery (Preoperative, yellow) and at necropsy (Postoperative, green). Parameters include ALB (albumin), ALP (alkaline phosphatase), ALT (alanine aminotransferase), BUN (blood urea nitrogen), CRE-P (creatinine), GGT (gamma-glutamyl transferase), T-Bil (total bilirubin), UA (uric acid), and TP (total protein), reflecting hepatic and renal function. No significant differences were observed between preoperative and postoperative values in any treatment group, indicating that neither Colloidose^®^ nor Surgiflo™ induced detectable systemic organ toxicity.


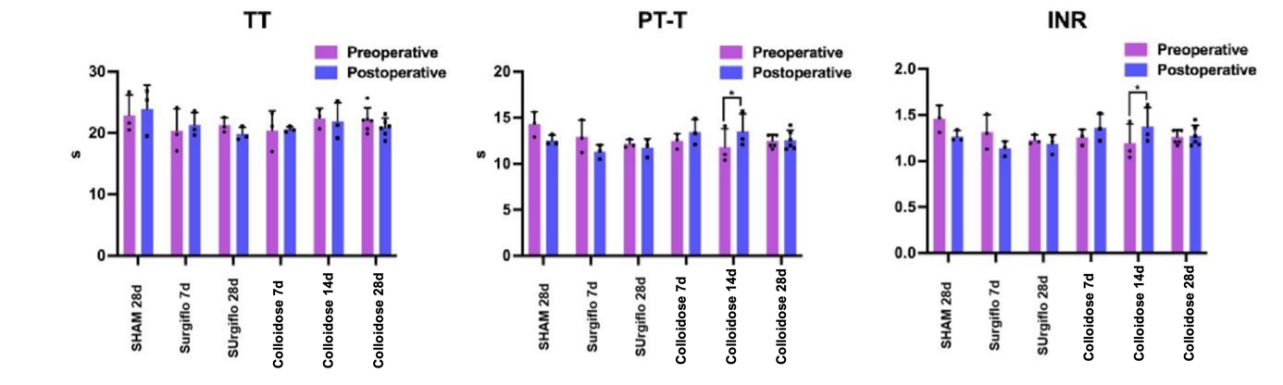


Figure S11. Coagulation profiles of Bama minipigs before surgery (preoperative) and at necropsy (postoperative) across different treatment groups. Parameters include thrombin time (TT), prothrombin time (PT), and international normalized ratio (INR). No clinically significant differences were observed between pre- and postoperative values within any group, indicating that neither Colloidose^®^ nor Surgiflo™ induced systemic coagulation abnormalities.


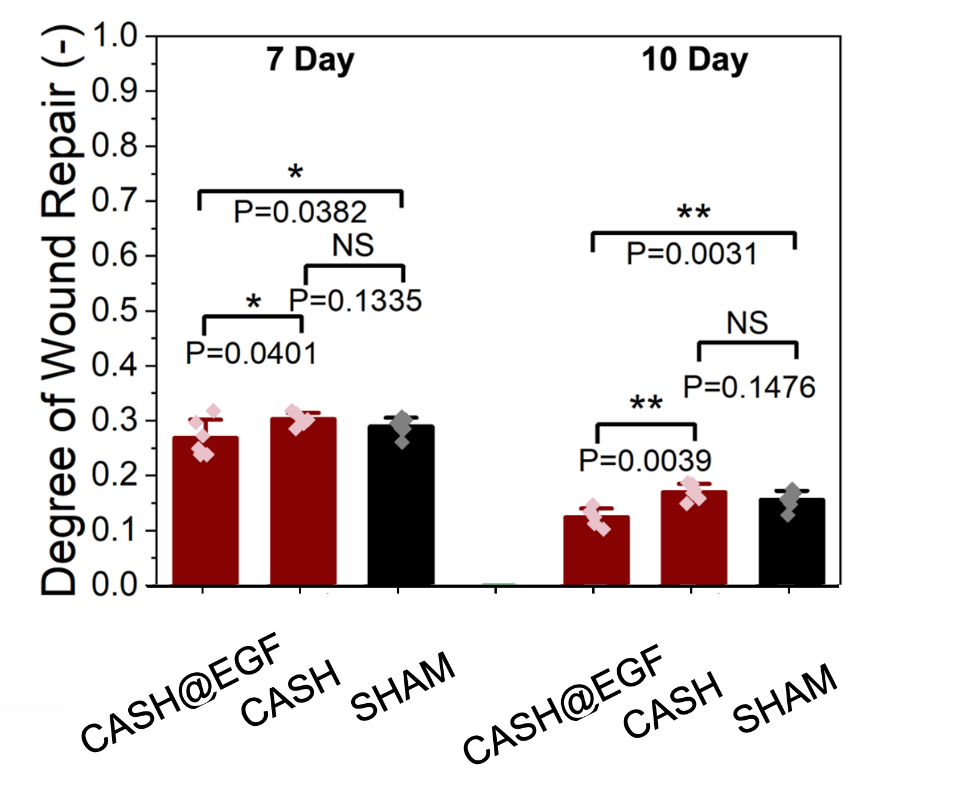


Figure S12. Quantitative analysis of the degree of wound repair in the CASH@EGF, CASH, and SHAM groups at Days 7 and 10 post-injury. Statistical analysis was performed using one-way ANOVA followed by Tukey’s multiple-comparison test. Exact P values are indicated in the figure; *P < 0.05, P < 0.01; NS, not significant.
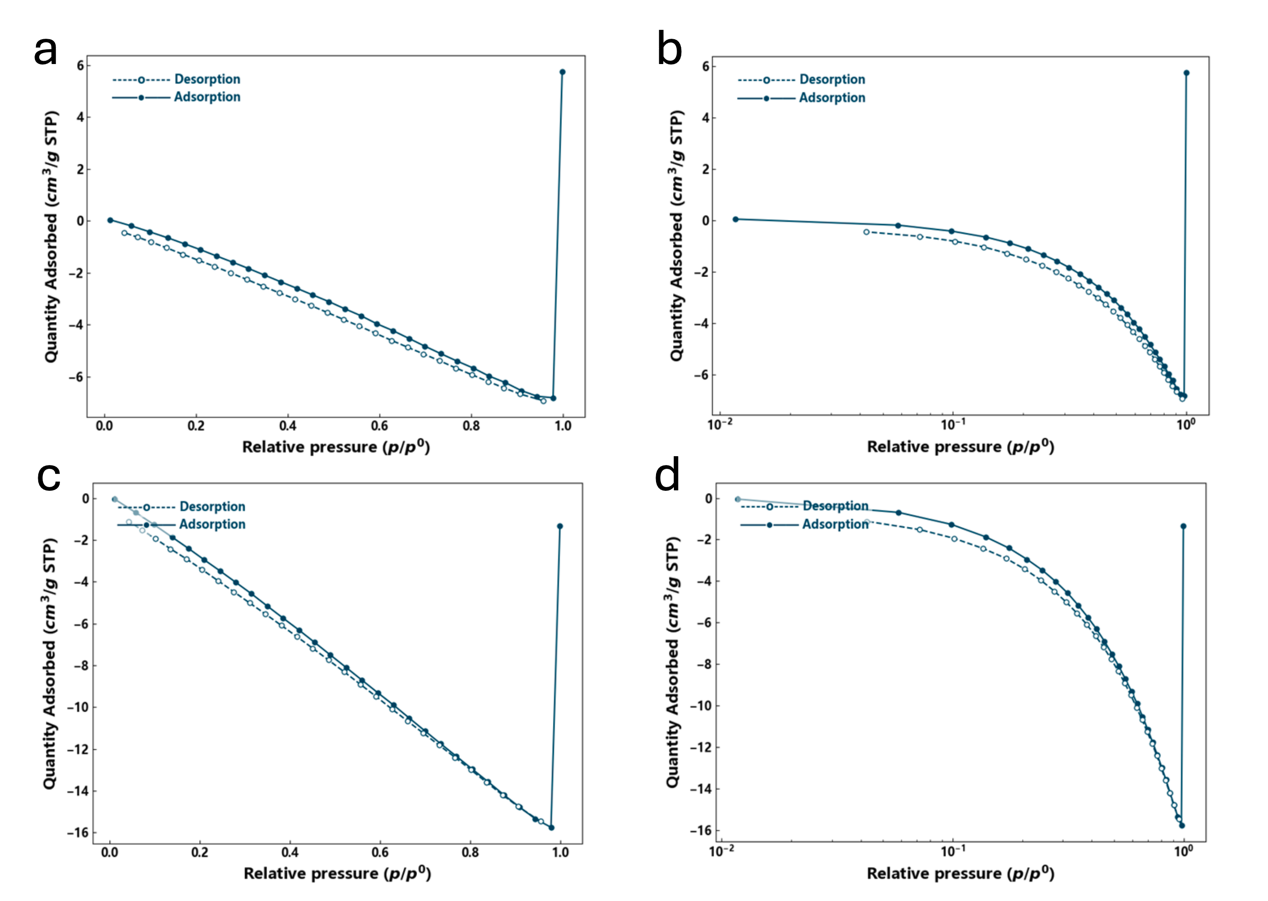


Figure S13. Nitrogen adsorption–desorption isotherms of (a, b) Colloidose^®^ and (c, d) Surgiflo™. (a, c) Isotherms plotted on a linear relative pressure (p/p₀) scale; (b, d) corresponding plots on a logarithmic p/p₀ scale. Adsorption and desorption branches are shown to illustrate the pore structure characteristics of the two matrices.

Table S2. Summary of biocompatibility and biological safety evaluation of Colloidose^®^ conducted in accordance with GB/T 16886 standards by accredited testing institutions.

| Test Category | Test Item | Standard | Key Result |
| --- | --- | --- | --- |
| National Medical Device Quality Supervision and Inspection Center, Jinan (China) | Rabbit pyrogen test | GB/T16886.11-2011 | Non-pyrogenic |
|  | Hemolysis test | GB/T16886.4-2003 | Hemolysis rate: 2% (<5% acceptance criterion) |
|  | Platelet test | GB/T16886.4-2003 | No significant effect on Platelet counts |
|  | Complement activation test | GB/T16886.4-2003 | Not an activator of the complement system |
|  | Hematology test | GB/T16886.4-2003 | No significant effect on RBC and WBC counts |
|  | Acute systemic toxicity test | GB/T16886.11-2011 | No observable acute systemic toxicity |
|  | In vitro cytotoxicity assay | GB/T16886.5-2017 | No potential cytotoxicity detected |
|  | Skin sensitization test | GB/T16886.10-2017 | No sensitization response observed |
|  | Intracutaneous reactivity test | GB/T16886.10-2017 | No erythema or edema observed |
|  | Bacterial reverse mutation test (Ames test) | GB/T16886.3-2019 | Negative |
|  | In vitro mouse lymphoma assay | GB/T16886.3-2019 | Negative |
|  | In vitro mammalian chromosomal aberration test | GB/T16886.3-2019 | No chromosomal aberration detected |
|  | Subchronic systemic toxicity test | GB/T16886.11-2011 | No systemic toxicity observed |
| Huatongwei International Inspection Co., Ltd. (China | Subacute systemic toxicity test | GB/T16886.11-2021 | No systemic toxicity observed |
| Jiangsu Kebiao Medical Testing Co., Ltd. (China) | Intramuscular implantation test | GB/T16886.11-2011 | Completely degraded at 4 weeks; no or minimal irritation |

Table S3. Baseline Demographic and Clinical Characteristics of Subjects in the Full Analysis Set (FAS).

| Project | | FAS | | | | |
| --- | --- | --- | --- | --- | --- | --- |
|  |  | Collodiose | Surgiflo | Testing method | Statistical measure | P Value |
| **age** |  |  |  |  |  |  |
| N(missing) |  | 174(0) | 174(0) | T-test | t=0.33 | 0.7447 |
| Mean±SD |  | 47.76±13.37 | 48.23±13.28 |  |  |  |
| median |  | 49 | 49 |  |  |  |
| Q1,Q3 |  | 37,58 | 40,58 |  |  |  |
| Min,Max |  | 18,74 | 18,4 |  |  |  |
| **Height** |  |  |  |  |  |  |
| N(missing) |  | 174(0) | 174(0) | T-test | t=0.74 | 0.4613 |
| Mean±SD |  | 163.51±8.33 | 162.89±7.34 |  |  |  |
| median |  | 162.5 | 162 |  |  |  |
| Q1,Q3 |  | 158,170 | 159,168 |  |  |  |
| Min,Max |  | 140,183 | 143,182 |  |  |  |
| **Weight** |  |  |  |  |  |  |
| N(missing) |  | 174(0) | 174(0) | T-test | t=0.27 | 0.7871 |
| Mean±SD |  | 66.48±13.12 | 66.84±11.41 |  |  |  |
| median |  | 64 | 66 |  |  |  |
| Q1,Q3 |  | 58,75 | 59,75 |  |  |  |
| Min,Max |  | 40,130 | 38,100 |  |  |  |
| **Body temperature** | |  |  |  |  |  |
| N(missing) |  | 174(0) | 174(0) | T-test | t=0.82 | 0.4108 |
| Mean±SD |  | 36.40±0.22 | 36.43±0.26 |  |  |  |
| median |  | 36.4 | 36.5 |  |  |  |
| Q1,Q3 |  | 36.2,36.5 | 36.3,36.5 |  |  |  |
| Min,Max |  | 36,37 | 35.5,37.2 |  |  |  |
| **Respiratory Rate (breaths per minute)** | | |  |  |  |  |
| N(missing) |  | 174(0) | 174(0) | T-test | t=2.15 | 0.0324 |
| Mean±SD |  | 18,89±1.01 | 19.12±1.03 |  |  |  |
| median |  | 19 | 19 |  |  |  |
| Q1,Q3 |  | 18,20 | 18,20 |  |  |  |
| Min,Max |  | 16,21 | 15,21 |  |  |  |
| **Diastolic Blood Pressure** | | |  |  |  |  |
| N(missing) |  | 172(2) | 172(2) | T-test | t=0.84 | 0.4278 |
| Mean±SD |  | 85.53±11.35 | 81.48±10.92 |  |  |  |
| median |  | 79 | 80 |  |  |  |
| Q1,Q3 |  | 74,88 | 73,89 |  |  |  |
| Min,Max |  | 55,115 | 57,116 |  |  |  |
| **Systolic Blood Pressure** | | |  |  |  |  |
| N(missing) |  | 172(2) | 172(2) | T-test | t=1.02 | 0.4033 |
| Mean±SD |  | 127.61±15.53 | 129.07±16.79 |  |  |  |
| median |  | 127 | 128 |  |  |  |
| Q1,Q3 |  | 117.5,135.3 | 118,139.50 |  |  |  |
| Min,Max |  | 87,178 | 90,183 |  |  |  |
| **Pulse Rate (beats per minute)** | | |  |  |  |  |
| Mean±SD |  | 79.38±10.55 | 78.29±9.28 | T-test | t=1.02 | 0.3085 |
| median |  | 78 | 78 |  |  |  |
| Q1,Q3 |  | 72,84 | 72,83 |  |  |  |
| Min,Max |  | 53,120 | 51,101 |  |  |  |
| **Gender** |  |  |  |  |  |  |
| Male |  | 59(33.91) | 49(28.16) | Chi-Square Test | 1.34 | 0.2466 |
| Female |  | 115(66.09) | 125(71.84) |  |  |  |
| Total |  | 174(100) | 174(100) |  |  |  |
| **Ethnicity** |  |  |  |  |  |  |
| Han Nationality | | 168(96.55) | 170(97.7) | Chi-Square Test | 0.41 | 0.521 |
| Other |  | 6(3.45) | 4(2.30) |  |  |  |
| Total |  | 174(100) | 174(100) |  |  |  |
| **Marital Status** | |  |  |  |  |  |
| Married |  | 157(90.23) | 159(91.38) | CMH test | 1.01 | 0.6036 |
| Unmarried |  | 14(8.05) | 14(8.05) |  |  |  |
| Other |  | 3(1.72) | 1(0.57) |  |  |  |
| Total |  | 174(100) | 174(100) |  |  |  |

Table S4. Hemostatic efficacy within 5 minutes in different analysis populations. FAS (Full Analysis Set) includes all animals analyzed according to the intention-to-treat principle. PPS (Per Protocol Set) includes animals who completed the study without major protocol deviations.

| Project | | FAS | | PPS | |
| --- | --- | --- | --- | --- | --- |
|  |  | Colloidose | Surgiflo | Colloidose | Surgiflo |
| **Hemostasis efficacy rate within 5 minutes** | | | |  |  |
| Effective |  | 174(100.00) | 174(100.00) | 174(100.00) | 172(100.00) |
| Ineffective |  | 0(0.00) | 0(0) | 0(0) | 0(0) |
| Total |  | 174(100.00) | 174(100.00) | 174(100.00) | 172(100.00) |
| **Comparison of therapeutic effects between two groups** | | | | |  |
| Statistical measure | | Z=0.00 | | Z=0.00 | |
| P value |  | 1 | | 1 | |
| **95% confidence interval for the difference in effectiveness between two groups** | | | | | |
| Newcombe-Wilson method | | (-0.0216,0.0216) | | (-0.0216,0.0218) | |
| Haldane method | | (0,0) | | (0,0.0001) | |
| Jeffreys-Perks method | | (-0.0112,0.0112) | | (-0.0112,0.0113) | |
| Brown-Li method | | (-0.0112,0.0112) | | (-0.0112,0.0113) | |

Table S5. Hemostatic efficacy within 3 minutes in different analysis populations. FAS (Full Analysis Set) includes all animals analyzed according to the intention-to-treat principle. PPS (Per Protocol Set) includes animals who completed the study without major protocol deviations.

| Project | | FAS | | PPS | |
| --- | --- | --- | --- | --- | --- |
|  |  | Colloidose | Surgiflo | Colloidose | Surgiflo |
| **Hemostasis efficacy rate within 3 minutes** | | | |  |  |
| Effective |  | 172(98.85) | 165(94.83) | 172(98.85) | 163(94.77) |
| Ineffective |  | 2(1.15) | 9(5.17) | 2(1.15) | 9(5.23) |
| Total |  | 174(100.00) | 174(100.00) | 174(100.00) | 172(100.00) |
| **Comparison of therapeutic effects between two groups** | | | | |  |
| Statistical measure | | Z=2.14 | | Z=2.16 | |
| P value |  | 0.0324 | | 0.0308 | |
| **95% confidence interval for the difference in effectiveness between two groups** | | | | | |
| Newcombe-Wilson method | | (0.0021,0.0847) | | (0.0025,0.0857) | |
| Haldane method | | (0.0035,0.0761) | | (0.0038,0.0771) | |
| Jeffreys-Perks method | | (0.002,0.0776) | | (0.0023,0.0786) | |
| Brown-Li method | | (0.002,0.078) | | (0.0023,0.079) | |

Table S6. Routine hematological parameters before and after treatment with Colloidose^®^ or Surgiflo™.

| Project | Screening period normal | | Abnormal during the screening period | | Missing data situation | | | Total |
| --- | --- | --- | --- | --- | --- | --- | --- | --- |
|  | Normal after treatment | Abnormal after treatment | Normal after treatment | Abnormal after treatment | Pre-treatment missing | Post-treatment missing | Missing before and after treatment |  |
| WBC |  |  |  |  |  |  |  |  |
| Colloidose | 127 | 26 | 14 | 5 | 0 | 2 | 0 | 174 |
| Surgiflo | 129 | 38 | 5 | 1 | 1 | 0 | 0 | 174 |
| RBC |  |  |  |  |  |  |  |  |
| Colloidose | 93 | 57 | 3 | 19 | 0 | 2 | 0 | 174 |
| Surgiflo | 94 | 55 | 3 | 21 | 1 | 0 | 0 | 174 |
| PLT |  |  |  |  |  |  |  |  |
| Colloidose | 143 | 9 | 9 | 11 | 0 | 2 | 0 | 174 |
| Surgiflo | 150 | 6 | 8 | 9 | 1 | 0 | 0 | 174 |
| LYM |  |  |  |  |  |  |  |  |
| Colloidose | 87 | 57 | 6 | 22 | 0 | 2 | 0 | 174 |
| Surgiflo | 86 | 65 | 10 | 12 | 1 | 0 | 0 | 174 |
| NEU |  |  |  |  |  |  |  |  |
| Colloidose | 109 | 41 | 13 | 9 | 0 | 2 | 0 | 174 |
| Surgiflo | 108 | 50 | 11 | 4 | 1 | 0 | 0 | 174 |
| CRP |  |  |  |  |  |  |  |  |
| Colloidose | 42 | 93 | 3 | 31 | 1 | 4 | 0 | 174 |
| Surgiflo | 38 | 102 | 1 | 28 | 4 | 1 | 0 | 174 |

Table S7. Comprehensive comparison of physicochemical properties, mechanical performance, and hemostatic efficacy between Colloidose^®^ and Surgiflo™.

| Property | Colloidose^®^ | Surgiflo™ | Notes |
| --- | --- | --- | --- |
| Particle Size | 293.4 ± 133.4 nm | ≈ 600 μm | Section 2.1, Fig. 1c–d |
| Surface Area (m²/g) | 24.8808 m²/g | 5.4637 m²/g | Table SXXX |
| Storage Modulus (G’, kPa) | 18.0 ± 0.6 kPa | 2.58 ± 0.19 kPa | Section 2.2, Fig. 2a–b |
| Self-healing effiency | > 90% recovery of initial G’ | < 95% recovery of initial G’ | Section 2.2, Fig. 2h–i |
| Stability in Aqueous Environment | maintains structural integrity in PBS and culture medium | Rapid disintegration and loss of cohesion | Section 2.1 & 2.4, Fig. 1j, Fig. 4a |
| Adhesive Strength | 2.02±0.57 kPa (CASH-15%) | 0.33±0.13 kPa | Section 2.3, Fig. 3d |
| Burst Pressure | 4.95 ± 0.83 kPa (CASH-15%) | 2.11 ± 0.25 kPa | Section 2.3, Fig. 3c |
| Hemostasis Time | 7.80 ± 4.32 s (CASH-15%) | 56.40 ± 24.88 s | Section 2.5, Fig. 5d |
| Hemostasis Success Rate (3 min) | 98.95% | 94.89% | Section 2.7, Table S2 |

Table S8. Distribution of clinical cases by participating hospitals and surgical departments.

| **Hospital** | **Gynecology** | **Orthopedics** | **General Surgery** | **Total** |
| --- | --- | --- | --- | --- |
| West China Hospital, Sichuan University | - | 38 | - | **38** |
| Affiliated Central Hospital of Dalian University of Technology | 20 | 19 | 19 | **58** |
| Taiyuan Central Hospital | 16 | 19 | 59 | **94** |
| Hebei PetroChina Central Hospital | 58 | 20 | - | **78** |
| Changzhi People's Hospital | 22 | 18 | 40 | **80** |
| **Total** | **116** | **114** | **118** | **348** |
